# Long non‐coding RNA SNHG14 induces trastuzumab resistance of breast cancer via regulating *PABPC1* expression through H3K27 acetylation

**DOI:** 10.1111/jcmm.13758

**Published:** 2018-07-31

**Authors:** Huaying Dong, Wei Wang, Shaowei Mo, Qiang Liu, Xin Chen, Ru Chen, Yu Zhang, Kejian Zou, Mulin Ye, Xionghui He, Fan Zhang, Jing Han, Jianguo Hu

**Affiliations:** ^1^ Department of General Surgery Hainan General Hospital Jinan University Haikou China; ^2^ Department of Science and Education Hainan Maternal and Child Health Hospital Haikou China; ^3^ Department of Pharmacy Hainan Medical University Haikou China; ^4^ Department of General Surgery The Frist Affiliated Hospital Chongqing Medical University Chongqing China; ^5^ Department of Obstetrics and Gynecology The Second Affiliated Hospital Chongqing Medical University Chongqing China

**Keywords:** breast cancer, H3K27 acetylation, PABPC1, SNHG14, trastuzumab resistance

## Abstract

Currently, resistance to trastuzumab, a human epidermal growth factor receptor 2 (HER2) inhibitor, has become one major obstacle for improving the clinical outcome of patients with advanced HER2^+^ breast cancer. While cell behaviour can be modulated by long non‐coding RNAs (lncRNAs), the contributions of lncRNAs in progression and trastuzumab resistance of breast cancer are largely unknown. To this end, the involvement and regulatory functions of lncRNA SNHG14 in human breast cancer were investigated. RT‐qPCR assay showed that SNHG14 was up‐regulated in breast cancer tissues and associated with trastuzumab response. Gain‐ and loss‐of‐function experiments revealed that overexpression of SNHG14 promotes cell proliferation, invasion and trastuzumab resistance, whereas knockdown of SNHG14 showed an opposite effect. *PABPC1* gene was identified as a downstream target of SNHG14, and *PABPC1* mediates the SNHG14‐induced oncogenic effects. More importantly, ChIP assays demonstrated that lncRNA SNHG14 may induce *PABPC1* expression through modulating H3K27 acetylation in the promoter of PABPC1 gene, thus resulting in the activation of Nrf2 signalling pathway. These data suggest that lncRNA SNHG14 promotes breast cancer tumorigenesis and trastuzumab resistance through regulating *PABPC1* expression through H3K27 acetylation. Therefore, SNHG14 may serve as a novel diagnostic and therapeutic target for breast cancer patients.

## 1 INTRODUCTION

Breast cancer has become a leading cause of cancer‐related deaths in the world and the most common cancer among women.^1^ The important reason of these deaths is distant metastasis and resistance to the currently available therapeutics.^2^ About 15%‐20% of breast cancer patients are accompanied with overexpression of human epidermal growth factor receptor 2 (HER2), ended up with poorer prognosis and survival.^3^, ^4^ Currently, therapy with anti‐HER2 mono‐antibody such as trastuzumab is applied to treat HER2‐positive breast cancer patients.^5^, ^6^ Trastuzumab is designed to target HER2 and silence its function, and is mostly used for early stage or metastatic gastric and breast cancer patients with positive HER2 mutations. However, trastuzumab might be effective at initial treatments, and resistance increases substantially after a period of exposure. In addition, there is a clear need for useful therapeutic biomarkers that can be used for predicting chemo‐response to trastuzumab treatment.^7^ Hence, it is urgent and meaningful to reveal the mechanism of trastuzumab resistance and find useful molecular markers and therapeutic targets for breast cancer patients.

With the advanced development of whole genome and transcriptome sequencing technologies and the ENCODE project, it is more and more clear that most of the genome DNA is represented in processed transcripts without or lacking of protein‐coding capacity.^8^ Long non‐coding RNAs (lncRNAs) are a recently discovered major class of non‐coding RNAs (ncRNAs) with more than 200 nucleotides in length.^9^ In recent years, emerging evidence indicates that they play important roles in regulating cellular and biological functions. LncRNAs can regulate gene expression at post‐transcriptional level via sponging microRNAs^10^ and modulate transcriptional gene silencing via the chromatin regulation.^11^, ^12^ Thus, the investigation of the role of lncRNAs in breast cancer could help with the understanding of tumorigenesis and the identification of novel diagnostic and therapeutic targets.

The lncRNAs work in a complicated way as critical regulator of epigenetic modulation, transcription and translation in a spatiotemporal manner.^13^, ^14^ SNHG14, alternatively named UBE3A‐ATS, is located on chromosome 15q11.2. SNHG14 can overlap with the entire UBE3A gene and promoter, thus inhibited the expression of UBE3A, causing neurogenetic disorders, such as Angelman syndrome.^15^ Recently, Liu et al^16^ demonstrated that SNHG14 could act as a ceRNA to promote initiation and progression of clear cell renal cell carcinoma by regulating N‐WASP protein. Another report from Wang et als^17^ showed that SNHG14 is a tumour suppressor gene by inhibiting proliferation and invasion in glioma. These contradictory conclusions lead us to identify the function of SNHG14 in breast cancer. Currently, however, the expression pattern, biological function and underlying mechanism of SNHG14 in breast cancer progression and trastuzumab resistance are largely unknown.

In this study, we has been suggested that lncRNA SNHG14 regulated breast cancer progression and resistance via regulating *PABPC1* expression through H3K27 acetylation. To verify this hypothesis, we determined the expression level of SNHG14 in breast cancer tissues and cell lines. By performing in vitro and in vivo experimental assays, we further investigated the functional relevance of SNHG14 with breast cancer progression.

## 2 MATERIALS AND METHODS

### 2.1 Patient samples

Primary cancer tissue and adjacent non‐cancerous tissue samples were collected from one cohort of 36 patients with breast cancer (male/female: 0/36, range of age (median): 35‐62 (46)), and another independent cohort of 62 breast cancer patients that received trastuzumab treatment (male/female: 0/62, range of age (median): 49‐81 (55)). All the patients were pathologically confirmed and the clinical tissue samples were collected before chemotherapy was started at Hainan General Hospital and The Second Affiliated Hospital of Chongqing Medical University. They were obtained during operation and immediately frozen at −80°C until RNA extraction. Written informed consents obtained from all patients were approved according to the guidelines revised by the Hainan General Hospital and The Second Affiliated Hospital of Chongqing Medical University.

### 2.2 Cell lines and reagents

The human breast cancer cell lines SKBR‐3 and BT474, which harbour HER2 activating mutations, were purchased from Chinese Type Culture Collection, Chinese Academy of Sciences (Shanghai, China). Both cell lines were cultured in RPMI 1640 medium (BioWhittaker, Lonza, USA) supplemented with 10 mmol/L Hepes, 1 mmol/L L‐glutamine, 100 U/mL penicillin/streptomycin (BioWittaker, Lonza) and heat inactivated 10% foetal bovine serum (FBS, Gibco) at 37°C in a humidified incubator with 5% CO_2_. Trastuzumab (Herceptin) was obtained from Roche (Basel, Switzerland) and dissolved in sterile water. Trastuzumab‐resistant SKBR‐3/Tr and BT474/Tr cells were obtained by continuous culture with 5 mg/mL trastuzumab for 6 months as previously reported,^11^, ^12^ and were cultured in RPMI 1640 medium with 250 μg/mL trastuzumab.

### 2.3 RNA oligoribonucleotides and cell transfection

The full‐length of lncRNA SNHG14 and the coding sequence of *PABPC1* were amplified, cloned into the lentivirus vector for retrovirus production with BT474 cells (Lv‐SNHG14 and Lv‐PABPC1) by GeneChem (Shanghai, China). Negative control vectors were also generated (Lv‐NC). The lentivirus vector containing shRNA sequence targeting *PABPC1* (sh‐PABPC1), SNHG14 (Lv‐SNHG14) or negative control vector (sh‐NC) was also amplified and cloned by GeneChem. All the vectors were labelled with green fluorescence protein (GFP). Transfection was carried out using Lipofectamine 3000 (Invitrogen, Carlsbad, CA, USA) following the manufacturer's instructions. Transfection efficiency was evaluated in every experiment by RT‐qPCR 24 hours later to ensure that cells were transfected. Functional experiments were then performed after sufficient transfection for 48 hours.

### 2.4 Reverse transcription‐quantitative polymerase chain reaction (RT‐qPCR)

RNA was reverse transcribed using the SuperScript III^®^ (Invitrogen) and then the obtained cDNAs were then quantified using RT‐qPCR assay labelled with SYBR (Takara Bio Company, Dalian, China) on Bio‐Rad CFX96 Sequence Detection System (Bio‐Rad company, Berkeley, CA, USA). The gene expression levels were normalized by GAPDH expression. RT‐qPCR results were analysed and expressed relative to CT (threshold cycle) values and then converted to fold changes. All the premier sequences were synthesized by RiboBio (Guangzhou, China), and their sequences are shown as follows: SNHG14 (Forward) 5′‐GGGTGTTTACGTAGACCAGAACC‐3′, (Reverse) 5′‐CTTCCAAAAGCCTTCTGCCTTAG‐3′; *PABPC1* (Forward) 5′‐AGCAAATGTTGGGTGAACGG‐3′, (Reverse) 5′‐ACCGGTGGCACTGT TAACTG‐3′; GAPDH (Forward) 5′‐GAAGGTGAAGGTCGGAGTC‐3′, (Reverse) 5′‐GAAGATGGTGATGGGA TTTC‐3′.

### 2.5 Cell viability assay

The altered cell viability after transfection was assayed using the CCK8 Kit (Dojindo, Rockville, MD, USA). In brief, cells were seeded into a 96‐well plate and then treated with silencing or overexpressing vectors for 48 hours. After, cells were treated with the CCK8 reagent and further cultured for 2 hours. The optical density at 450 nm was measured with a spectrophotometer (Thermo Electron Corporation, MA, USA). The percentage of the control samples of each cell line was calculated thereafter.

### 2.6 Cell migration and invasion assays

Cell migration was evaluated by performing wound healing assay. Wounds were scratched on the monolayer of cells using 20 μL pipette tips. Plates were washed once with fresh medium to remove non‐adherent cells after the cells had been cultured for 48 h and then photographed. Cell invasion ability was tested using transwell invasion assay. Briefly, 100 μL Matrigel (BD, USA) was firstly added onto the bottom of the transwell chamber (24‐well insert; 8‐mm pore size, Corning Costar Corp), then 1 × 10^5^ cells in reduced serum medium (Opti‐MEM, Gibco) were placed on the coated membrane in the chamber. Migrated cells on the permeable membrane were fixed and then stained with crystal violet.

### 2.7 Chromatin immunoprecipitation (ChIP)

ChIP was performed with the EZ ChIP™ Chromatin Immunoprecipitation Kit (Millipore, Burlington, MA, USA) according to the manufacturer's protocol. Briefly, cross‐linked chromatin was sonicated into 200‐1000 bp fragments. The chromatin was immunoprecipitated using anti‐H3K27ac antibodies (Abcam, ab4729, Cambridge, MA, USA).

### 2.8 Immunofluorescence

Cells were fixed in 4% formaldehyde for 15 minutes and then washed with PBS. The fixed cells were treated with pepsin and dehydrated through ethanol, and further permeabilized in Triton X‐100 (Sigma‐Aldrich) for 20 minutes. Goat serum was used for blocked, and then, cells were incubated with anti‐Ki67 antibody (Abcam, ab15580, 1:500, Cambridge, MA, USA) for overnight at 4°C and then incubated with the appropriate rhodamine‐conjugated secondary antibody for 1 hour. The cells were then washed and incubated with DAPI (Invitrogen) for nuclear staining. The cells were visualized for immunofluorescence with a fluorescence microscopy (DMI4000B, Leica).

### 2.9 Fluorescence in situ hybridization analysis (FISH)

Nuclear and cytosolic fraction separation was performed with a PARIS kit (Life Technologies), and RNA FISH probes were designed and synthesized by Bogu according to the manufacturer's instructions. Briefly, cells were fixed in 4% formaldehyde for 15 minutes and then washed with PBS. The fixed cells were treated with pepsin and dehydrated through ethanol. The air‐dried cells were incubated further with 40 nmol/L of the FISH probe in hybridization buffer. After hybridization, the slide was washed, dehydrated and mounted with Prolong Gold Antifade Reagent with DAPI for detection. The slides were visualized for immunofluorescence with a fluorescence microscopy (DMI4000B, Leica).

### 2.10 TUNEL assay

TUNEL staining was performed to evaluate cell apoptosis. In brief, cells were fixed using 4% formaldehyde followed by staining with TUNEL kit according to the manufacturer's instructions (Vazyme, TUNEL Bright‐Red Apoptosis Detection Kit, A113). TUNEL‐positive cells were counted under fluorescence microscopy (DMI4000B, Leica).

### 2.11 Immunohistochemistry analysis

Immunohistochemical staining was performed on 4‐μm‐thick TMA slides. Briefly, the slides were deparaffinized and antigen retrieval was then performed in a steam cooker for 1.5 minutes in 1 mmol/L EDTA. Rabbit anti‐PABPC1 antibody (#4992, Cell Signaling Technology, Beverly, MA, USA) at 1:150 dilution was used for culture over night at 4 □. Universal secondary antibody (DAKO) was applied for 15 minutes at room temperature. Diaminobenzidine or 3‐amino‐9‐ethylcarbazole was used as chromogens and slides were counterstained with haematoxylin before mounting.

### 2.12 In vivo tumorigenesis assay

Male BALB/C nude mice (6 weeks of age) were purchased from Shanghai SIPPR‐BK Laboratory Animal Co. Ltd. (Shanghai, China) and maintained in microisolator cages. 1 × 10^7^ BT474 cells transfected with Lv‐SNHG14 or Lv‐NC were suspended in 110 μL of serum‐free RPMI and then injected subcutaneously in the flank. Trastuzumab was dissolved in 1% Tween‐80. When tumours were palpable, the mice were randomized into the trastuzumab treatment groups or control groups. Treatment lasts for 4 weeks until the xenograft tumour was stripped and the mass was calculated.

### 2.13 Western blots and antibodies

Cell lysates were prepared with RIPA buffer containing protease inhibitors (Sigma‐Aldrich). Membranes were incubated overnight at 4°C with a 1:1000 solution of antibodies. A secondary antibody was then used for immunostaining for one hour at room temperature. The primary antibodies used here are anti‐PABPC1 antibody (Abcam, ab186533), anti‐Nrf2 antibody (Abcam, ab62352), anti‐HO‐1 antibody (Abcam, ab68477) and anti‐GAPDH antibody (Abcam, ab9485).

### 2.14 Statistical analysis

Measurement data were shown as median value. Count dates were described as frequency and examined using Fisher's exact test. The Mann‐Whitney *U* test or Kruskal‐Wallis test was used for evaluating the difference among different clinical or cell‐treated groups. Receiver operating characteristic (ROC) curves were established using MedCalc 9.3.9.0 (MedCalc, Mariakerke, Belgium), and the area under the ROC curve (AUC) was used as an accuracy index for evaluating the predictive performance of SNHG14. All statistical analyses were performed with SPSS 17.0 software (SPSS Incorporation, Chicago, IL, USA). Error bars in figures represent SD (Standard Deviation). The results were considered statistically significant at *P* < .05.

## 3 RESULTS

### 3.1 lncRNA SNHG14 is up‐regulated in breast cancer and can serve as a diagnostic indicator

**Figure 1:**
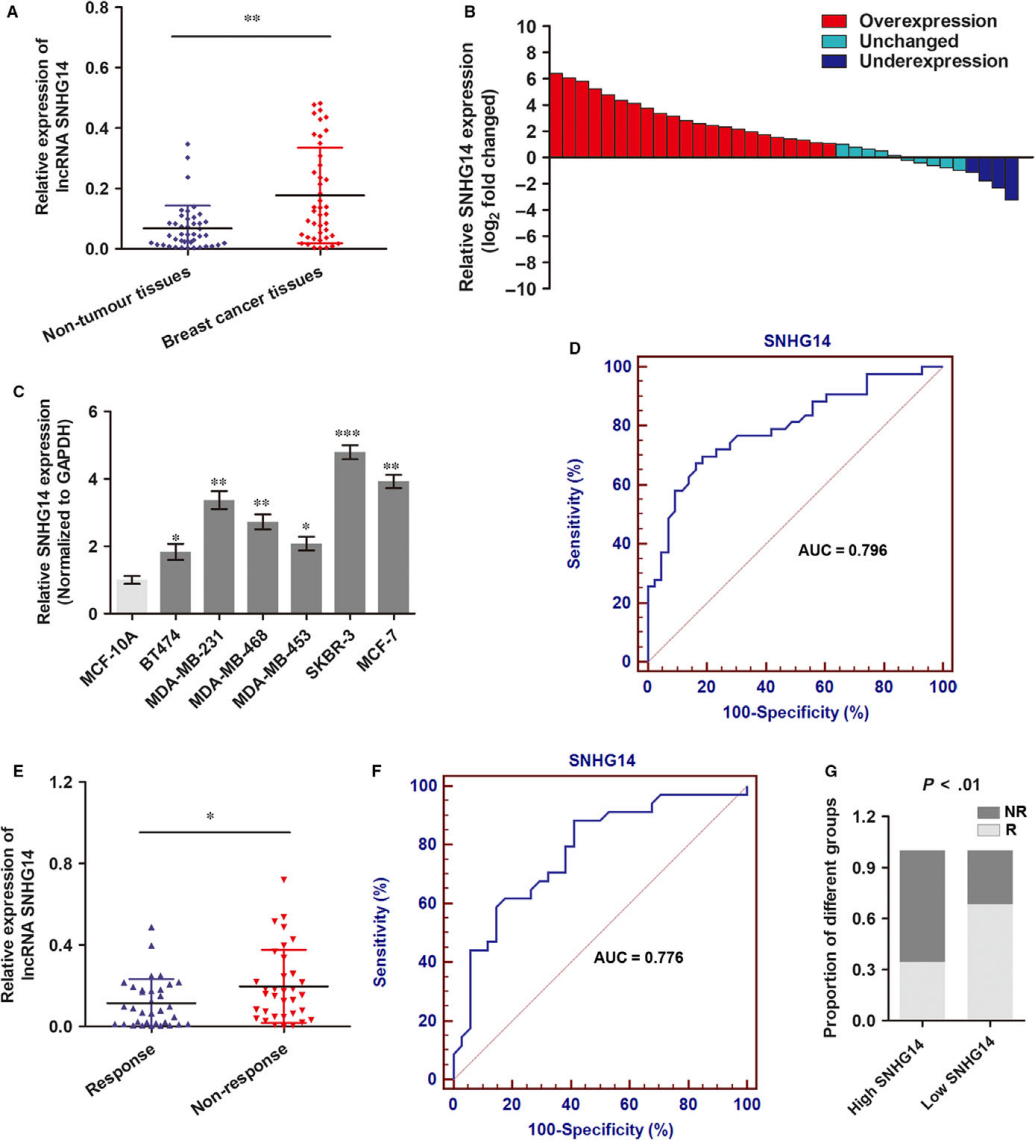
lncRNA SNHG14 is up‐regulated in breast cancer and can serve as a diagnostic indicator. A, RT‐qPCR was used to detect the expression of lncRNA SNHG14 in 36 paired breast cancer tissues and adjacent non‐tumour tissues. B, SNHG14 expression level was analysed in 36 primary breast cancer tissues and expressed as log_2_ fold change (cancer/normal), and the log_2_ fold changes were presented as follows: >1, overexpression (22 cases); <1, underexpression (4 cases); the remainder were defined as unchanged (10 cases). C, The expression level of SNHG14 was detected via RT‐qPCR in breast cancer cells. D, ROC curve was drawn to show the ability of SNHG14 in differentiating breast cancer patients from healthy individuals. E, RT‐qPCR was used to detect the expression of SNHG14 in 33 responding and 29 non‐responding patients who received trastuzumab therapy. F, ROC curve was drawn to show the ability of SNHG14 in differentiating responding patients from non‐responding patients. G, The proportion of patients that showed resistance to trastuzumab therapy was significantly higher in high SNHG14 expressing groups than in low expression group. *P <* *.05, **P <* *.01

To investigate whether the expression of SNHG14 is altered in breast cancer, we detected the expression of SNHG14 in 36 breast cancer tissues and paired adjacent non‐tumour tissues. As shown in Figure 1A, a significant elevated expression of SNHG14 was identified in breast cancer tissues when compared to matched non‐tumour tissues. In addition, 61.1% (22 of 36) cases showed more than 2‐fold higher level of SNHG14 in breast cancer tissues in contrast to paired normal tissues (Figure 1B). We then identified the expression level of SNHG14 in breast cancer cells and found that SNHG14 was up‐regulated in six breast cancer cells when compared to normal breast epithelium MCF‐10A cells (Figure 1C). Based on the up‐regulation model of SNHG14 in breast cancer, we investigated the diagnostic potential of SNHG14 in differentiating breast cancer patients from healthy group. ROC analysis showed an area under the curve (AUC) of 0.796, with a diagnostic sensitivity and specificity reaching 69.8% and 81.4% (95% CI = 0.695‐0.875) for SNHG14 expression in tissues (Figure 1D).

To determine the expression of SNHG14 in trastuzumab‐treated patients, we collected 62 cancer tissues from advanced HER2^+^ breast cancer patients who received single trastuzumab treatment. Patients were divided into responding (CR + PR, 33 patients) and non‐responding (SD + PD, 29 patients) groups according to the Response Evaluation Criteria In Solid Tumors (RECIST, version 1.1).^18^ RT‐qPCR showed that SNHG14 was up‐regulated in patients who did not respond to trastuzumab treatment than those who showed response to trastuzumab therapy (Figure 1E). We then investigated the diagnostic potential of SNHG14 by establishing a ROC curve. As shown in Figure 1F, the area under the curve (AUC), diagnostic sensitivity and specificity for differentiating responding and non‐responding patients reached 0.776, 67.65% and 70.59% with the established cut‐offs (0.033 at highest AUC), respectively. Under these stratification criteria (0.033), patients were divided into a low and a high SNHG14 expression groups, and the proportion of patients not responding to chemotherapy was significantly higher in the high SNHG14 expression group than in the low expression group (Figure 1G). Taken together, our clinical data indicate that SNHG14 may be a promising diagnostic marker for breast cancer patients.

### 3.2 lncRNA SNHG14 promotes proliferation and invasion of breast cancer cells in vitro

**Figure 2:**
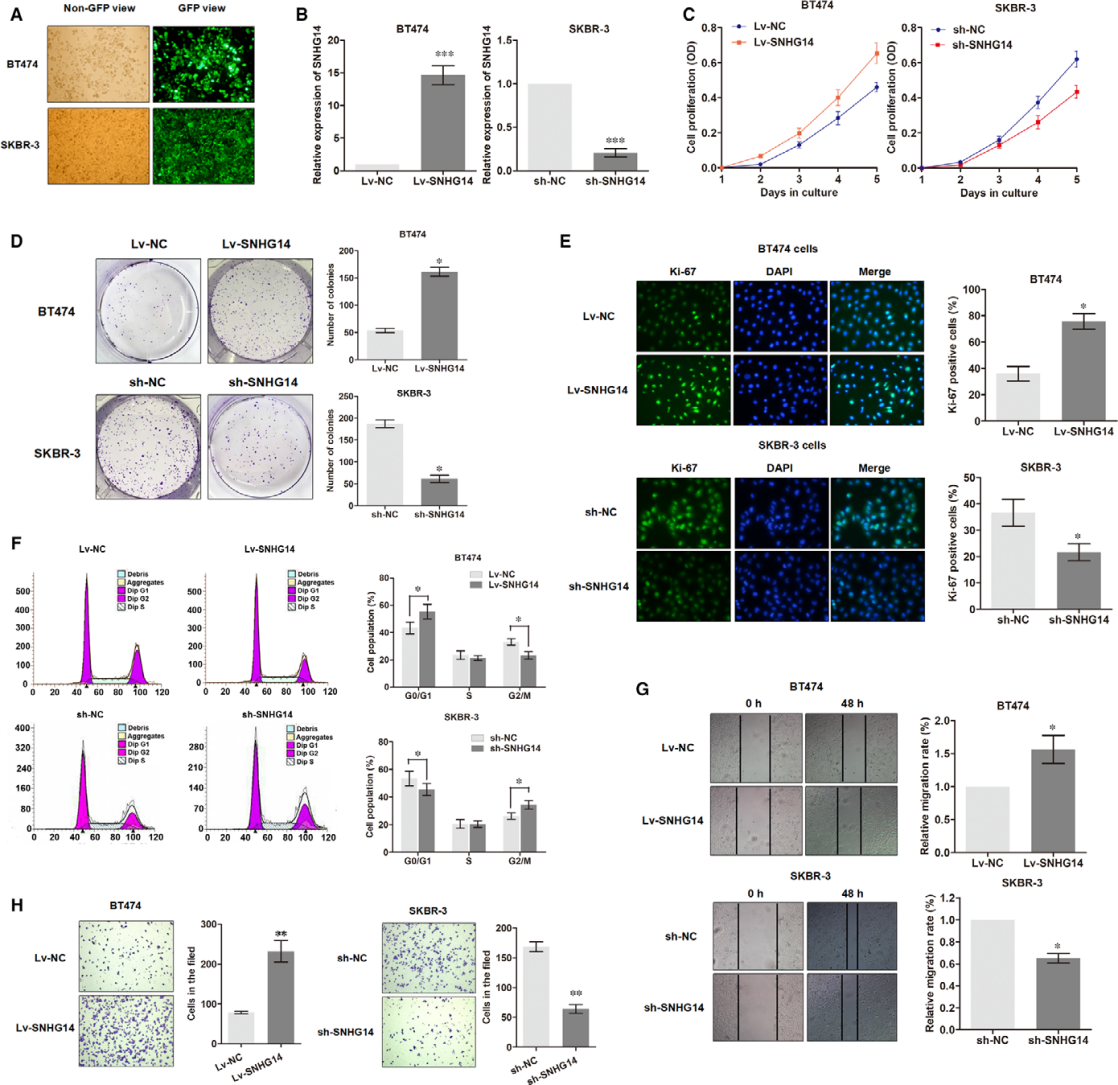
lncRNA SNHG14 promotes proliferation, migration and invasion of breast cancer cells. A, The oligonucleotides labelled with GFP green fluorescence were transfected as described in Methods. B, Transfection efficiency was identified by detecting SNHG14 expression via RT‐qPCR. C, CCK8 assay showed the functional effect of SNHG14 on cell proliferation of breast cancer cells. D, Colony formation assay was used to determine the functional role of SNHG14 for colony formation. E, Immunofluorescence analysis of Ki‐67 expression in breast cancer cells after infection of respective oligonucleotides. F, FACS cell cycle analysis of cells overexpressed or knockdown of SNHG14. The distribution of the cell cycle was shown in the graphs. G,H, The effects of SNHG14 on cell migration and invasion were determined using wound‐heal assay and transwell invasion assay. *P <* *.05, **P <* *.01, ***P <* *.001

We then investigated the functional role of SNHG14 in breast cancer progression and trastuzumab resistance using two HER2+ cell lines, SKBR‐3 and BT474. According to the expression of SNHG14 in breast cancer cells, among which SKBR‐3 showed the highest level while BT474 indicating a lowest endogenous expression, we constructed SNHG14 overexpression model using BT474 cells and SNHG14 knockdown model using SKBR‐3 cells (Figure 2A,B). According to the results from CCK8 assay, we found that BT474 cells overexpressed with SNHG14 showed significantly elevated cell proliferation compared to negative controls, while knockdown of SNHG14 in SKBR‐3 cells caused decreased cell growth (Figure 2C). Further in colony formation assay, the number of formed colonies was much higher in Lv‐SNHG14‐BT474 cells than Lv‐NC‐BT474 cells; however, a suppressed colony formation ability was identified in sh‐SNHG14‐SKBR‐3 cells when compared to sh‐NC‐SKBR‐3 cells (Figure 2D). To confirm the effect of SNHG14 on cell proliferation, we detected the Ki‐67 expression level by immunofluorescence assay. As expected, enhanced SNHG14 promoted Ki‐67 expression, whereas SNHG14 knockdown dramatically silenced the level of Ki‐67 (Figure 2E). Next, FACS cell cycle assay showed an increased population in G0/G1 phase and a decreased population in G2/M phase in SNHG14‐overexpressed BT474 cells, whereas knockdown of SNHG14 suggested an opposite effect in SKBR‐3 cells (Figure 2F).

Subsequently, we determined whether lncRNA SNHG14 had effects on cell migration and invasion. Wound healing assay indicated that overexpression of SNHG14 promoted cell migration, while knockdown of SNHG14 suppressed the migratory ability (Figure 2G). Next, transwell assay also showed that SNHG14 promoted breast cancer cell invasion (Figure 2H). To this end, we demonstrated that SNHG14 played an oncogenic role in breast cancer progression.

### 3.3 lncRNA SNHG14 induces trastuzumab resistance of breast cancer cells in vitro

**Figure 3:**
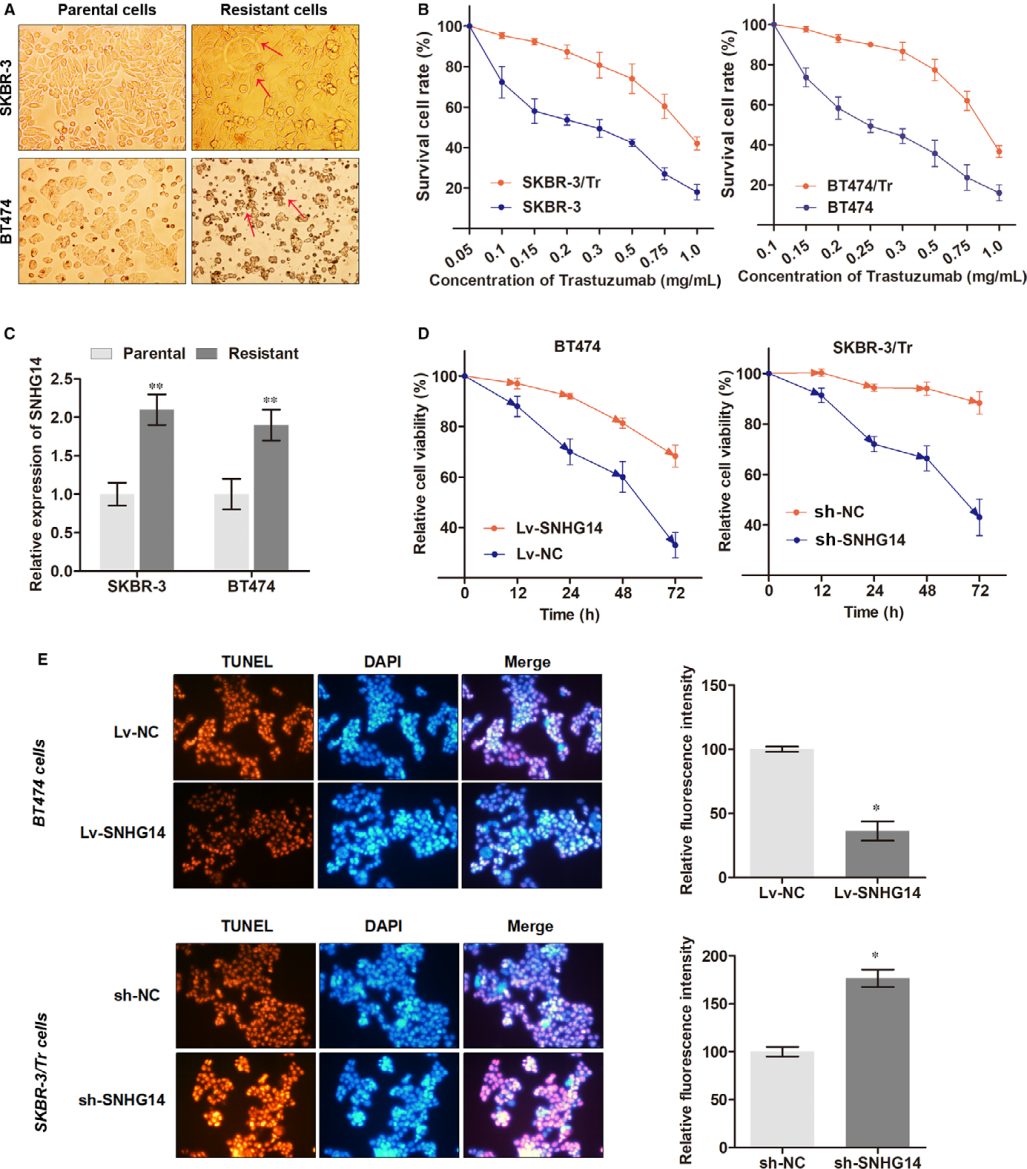
lncRNA SNHG14 promotes trastuzumab resistance of breast cancer. A, Trastuzumab‐resistant cell lines, SKBR‐3/Tr and BT474/Tr, were presented with specific morphologic changes in contrast to parental cells. Arrows show the specific changes compared to parental cells. B, The IC_50_ value of trastuzumab was detected for parental and resistant cell lines by incubating at indicated concentration of trastuzumab for 48 h. C, SNHG14 expression was detected via RT‐qPCR in breast cancer parental and trastuzumab‐resistant cell lines. D, Cell viability was evaluated in cells treated with trastuzumab (7.0 mg/mL) for 72 h after transfection of respective oligonucleotides. E, TUNEL assay was performed after the transfected cells were treated with trastuzumab at the concentration of 7.0 mg/mL for 36. *P < .05, **P < .01

Next, we investigated the functional relevance with trastuzumab in breast cancer cells. Two trastuzumab‐resistant sublines derived from HER2^+^ parental cell lines SKBR‐3 and BT474 were established (SKBR‐3/Tr and BT474/Tr, respectively). As shown in Figure 3A, the built trastuzumab‐resistant cells induced specific morphologic changes compared to parental cells, including loss of cell polarity, increased intercellular separation and increased formation of pseudopodia. The concentration‐effect curves suggest that the IC_50_ of trastuzumab (48 hours) for SKBR‐3/Tr cells is 0.83 mg/mL whereas 0.29 mg/mL for parental cells, which means that the SKBR‐3/Tr was 2.86 times the ability of trastuzumab resistance of SKBR‐3 parental cells. Similarly, the BT474/Tr cell was 3.29 times the ability of trastuzumab resistance of BT474 parental cells (0.79/0.24, Figure 3B).

The expression level of SNHG14 in trastuzumab‐resistant cells were also determined. As shown in Figure 3C, SNHG14 was up‐regulated in resistant cell lines in contrast to their parental cell lines. In addition, overexpression of SNHG14 partially abrogated the effects of trastuzumab on cell viability in BT474 cells whereas knockdown of SNHG14 in SKBR‐3/Tr promoted the trastuzumab‐induced cell cytotoxicity (Figure 3D). TUNEL assay was then used to detect whether SNHG14 influenced the nuclear apoptosis caused by trastuzumab. As expected, overexpression of SNHG14 suppressed the trastuzumab‐induced cell apoptosis in BT474 parental cells whereas knockdown of SNHG14 enhanced the apoptosis caused by trastuzumab in SKBR‐3/Tr cells (Figure 3E).

### 3.4 *PABPC1* is a downstream target of lncRNA SNHG14 function in breast cancer

**Figure 4:**
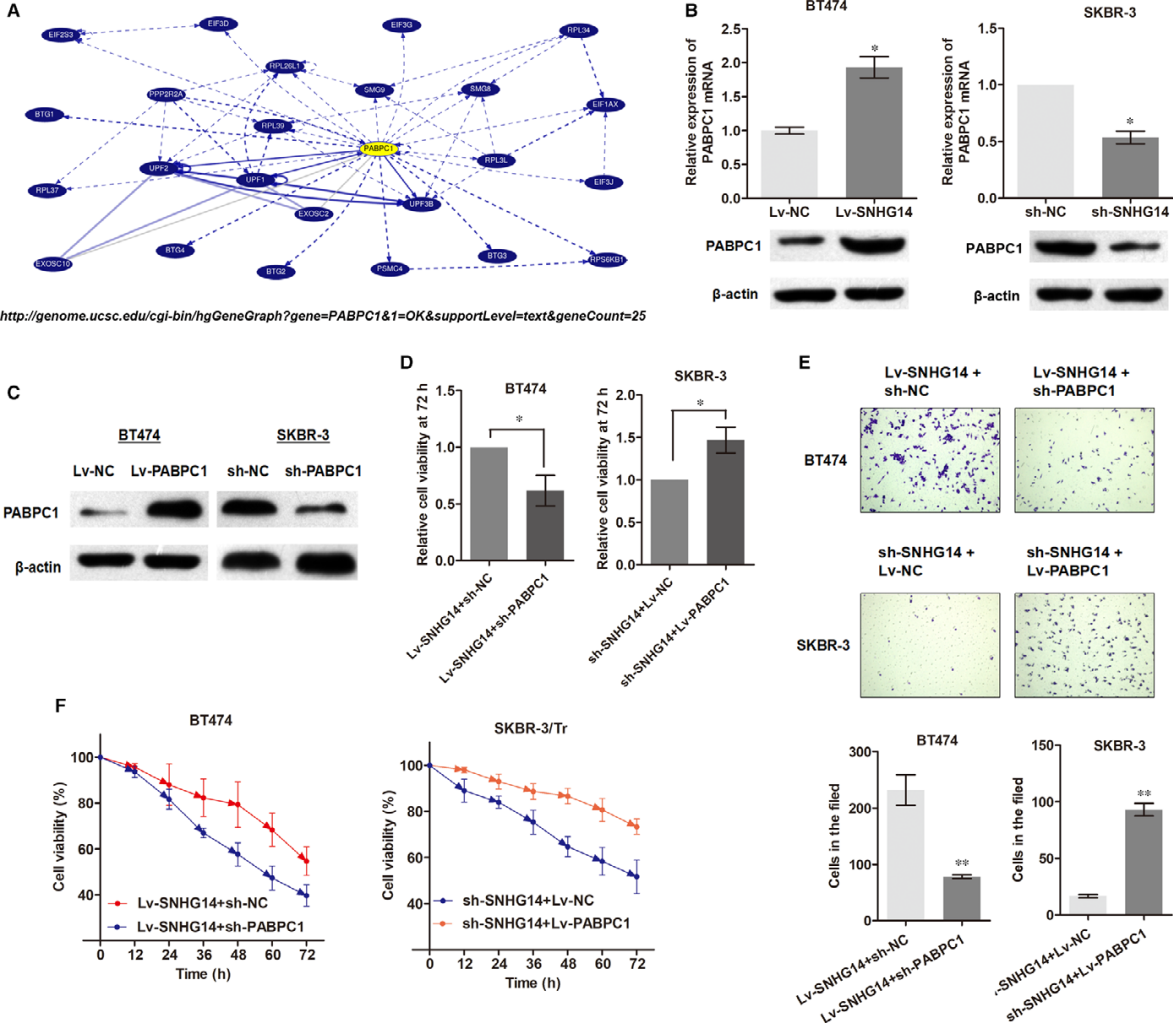
*PABPC1* is a downstream target of lncRNA SNHG14 function in breast cancer. A, Schematic diagram of network of *PABPC1* based on ENCODE database (http://genome.ucsc.edu). B, *PABPC1* was positively regulated by SNHG14 at both transcript and protein levels in breast cancer cells. C, PABPC1 expression was detected via Western blot after transfection of sh‐PABPC1 or Lv‐PABPC1. D, Knockdown of *PABPC1* dramatically abrogated the effects of Lv‐SNHG14 on cell proliferation, whereas overexpression of *PABPC1* reversed the effect induced by sh‐SNHG14. E, *PABPC1* knockdown or overexpression vector reversed the Lv‐SNHG14‐ or sh‐SNHG14‐indued effects of cell invasion, respectively. F, Cells of different groups were treated with trastuzumab (7.0 mg/mL) for 48 h, then the cell viability was determined using CCK8 assay. *P <* *.05, **P <* *.01

Based on the understanding of the pathologic role of SNHG14, we continued to determine the underlying functional mechanisms. RNA‐pull down experiments were performed followed by mass spectrometry to search for the SNHG14‐associated proteins in breast cancer cells. As shown in Table S1, a list of correlative SNHG14‐associated proteins was identified, among which Poly(A) Binding Protein Cytoplasmic 1 (*PABPC1*) was chosen as a potential target of SNHG14 according to our preliminary evaluation. *PABPC1* is a member of *PABPC* family, which binds specifically to the poly(A) tail of mRNA in cytoplasmic, is required for poly(A) shortening, ribosome recruitment and translation initiation,^19^ and critical for gene regulation by interacting with other RNA‐binding proteins (Figure 4A). Here, our in vitro experiments showed that overexpression of SNHG14 up‐regulated the expression of *PABPC1* at both mRNA and protein levels, whereas knockdown of SNHG14 down‐regulated the expression level (Figure 4B).

To further investigate whether *PABPC1* is a functional target of SNHG14, we constructed PABPC1‐overexpression vector and PABPC1‐knockdown vector (Figure 4C). Then, we modulated the expression of endogenous SNHG14 and *PABPC1* simultaneously. CCK8 assay clearly showed that knockdown of *PABPC1* dramatically abrogated the effects of Lv‐SNHG14 on cell proliferation, and overexpression of *PABPC1* reversed the effect induced by sh‐SNHG14 (Figure 4D). Similarly, *PABPC1* knockdown or overexpression vector reversed the Lv‐SNHG14‐ or sh‐SNHG14‐indued effects of cell invasion, respectively (Figure 4E). More importantly, co‐expression of Lv‐PABPC1 reversed the effects of down‐regulated SNHG14 in SKBR‐3/Tr cells whereas cotransfection of sh‐PABPC1 abrogated the resistance induced by Lv‐SNHG14 in BT474 parental cells (Figure 4F). To conclude, lncRNA SNHG14 may promote breast cancer progression and trastuzumab resistance via binding to *PABPC1* gene.

### 3.5 lncRNA SNHG14 induces an up‐regulation of *PABPC1* by modulating H3K27 acetylation of the promoter region of *PABPC1*

**Figure 5:**
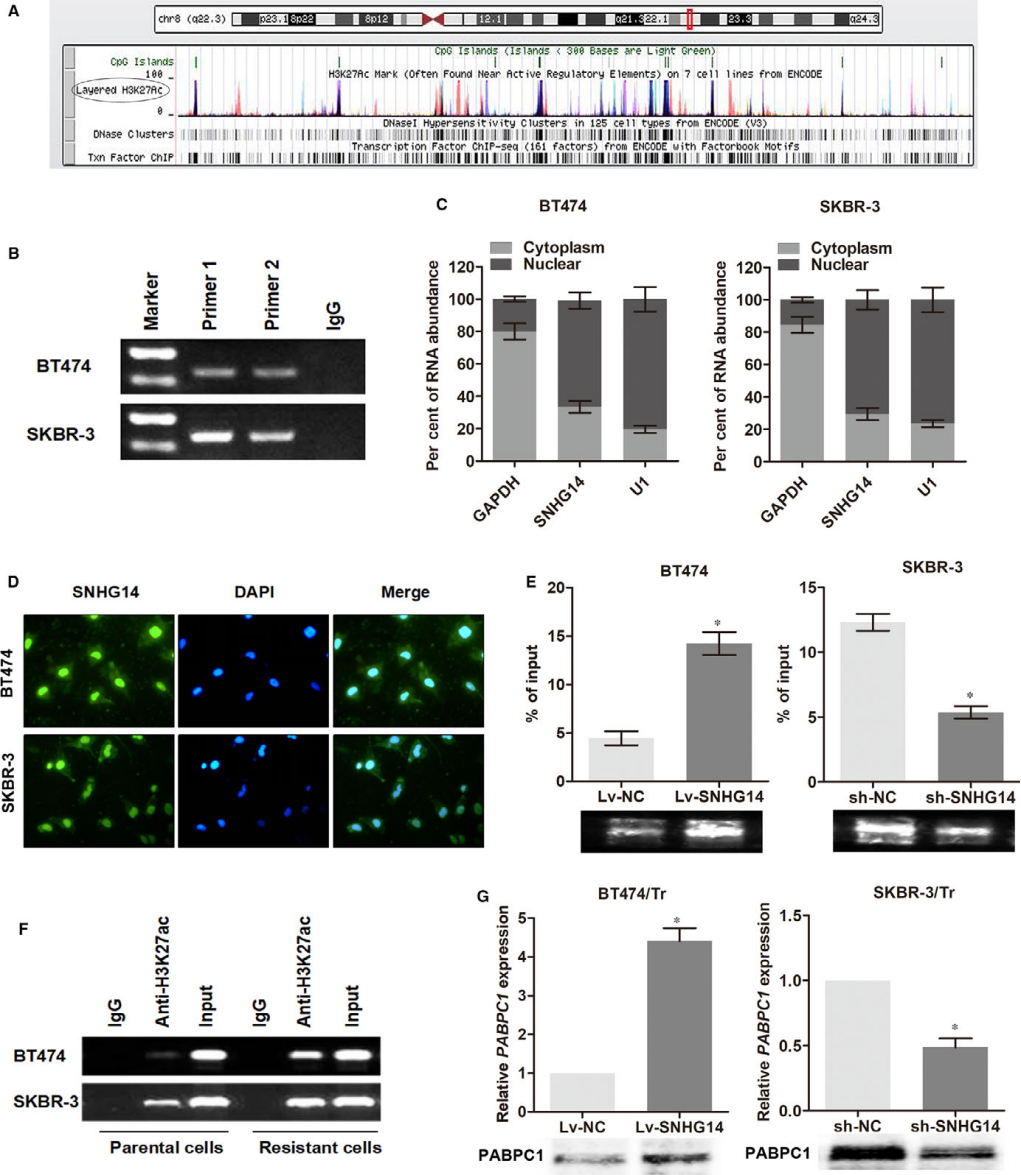
lncRNA SNHG14 induces an up‐regulation of *PABPC1* by modulating H3K27 acetylation of the promoter region of *PABPC1*. A, The genome bioinformatics analysis showed that the promoter of *PABPC1* had a high enrichment of H3K27ac. B, ChIP assay demonstrated that H3K27 acetylation occurred in the promoter of *PABPC1* in breast cancer cell lines using two primers. C, The expression level of SNHG14 in nuclear and cytoplasm of breast cancer cells. U1 (nuclear retained) and GAPDH (exported to cytoplasm) were used as controls. D, FISH analysis of the subcellular location of SNHG14 with specific probe in breast cancer cells. E, ChIP assay showed that SNHG14 positively regulated the enrichment of H3K27ac at *PABPC1* promoter. F, ChIP assay demonstrated that the enrichment of H3K27ac was higher in trastuzumab‐resistant cells than in parental cells. G, *PABPC1* was up‐regulated by Lv‐SNHG14 in BT474/Tr cells and down‐regulated by sh‐SNHG14 in SKBR‐3/Tr cells compared with the controls. *P <* *.05

To further understand the regulation of *PABPC1* by SNHG14, we explored the probable mechanisms by analysis of ENCODE database (http://genome.ucsc.edu/). As shown in Figure 5A, there was high level of enrichment of H3K27ac in the promoter region of *PABPC1*, indicating that histone acetylation might participate in the expression of *PABPC1* in transcriptional regulation. To test this hypothesis, we performed ChIP assay using SKBR‐3 and BT474 cells. As shown in Figure 5B, the enrichment of H3K27ac at promoter of *PABPC1* gene was identified in both cell lines. To further investigate whether lncRNA SNHG14 regulates the H3K27 acetylation at *PABPC1* promoter, we sublocated the expression of SNHG14 in breast cancer cells. RT‐qPCR analysis of nuclear and cytoplasmic lncRNA showed that lncRNA SNHG14 was enriched in nuclear section of both SKBR‐3 and BT474 cells (Figure 5C). FISH assay with specific probe of SNHG14 further confirmed that SNHG14 was mainly distributed in the nuclear section of both cells (Figure 5D). Then, we detected the enrichment of H3K27ac in the promoter of *PABPC1* by ChIP assay. As expected, up‐regulation of SNHG14 in BT474 cells increased the enrichment of H3K27ac in *PABPC1,* whereas knockdown of SNHG14 resulted in a decreased enrichment in SKBR‐3 cells (Figure 5E).

Next, we investigated whether acetylation of H3K27 is critical for trastuzumab resistance. ChIP assay showed that there was an elevated enrichment of H3K27ac of *PABPC1* in trastuzumab‐resistant cells when compared to the respective parental cells (Figure 5F). As enhanced acetylation of H3K27 was an important agonist for gene expression, we detected the expression of *PABPC1* upon transfection of SNHG14 in trastuzumab‐resistant cells and found that both mRNA and protein levels of *PABPC1* were up‐regulated by Lv‐SNHG14 in BT474/Tr cells and down‐regulated by sh‐SNHG14 in SKBR‐3/Tr cells compared with the controls (Figure 5G). Collectively, we draw a conclusion that lncRNA SNHG14 regulates *PABPC1* expression in breast cancer via modulation of H3K27 acetylation at promoter of *PABPC1*.

### 3.6 lncRNA SNHG14 facilitates tumorigenesis and trastuzumab resistance in vivo

**Figure 6:**
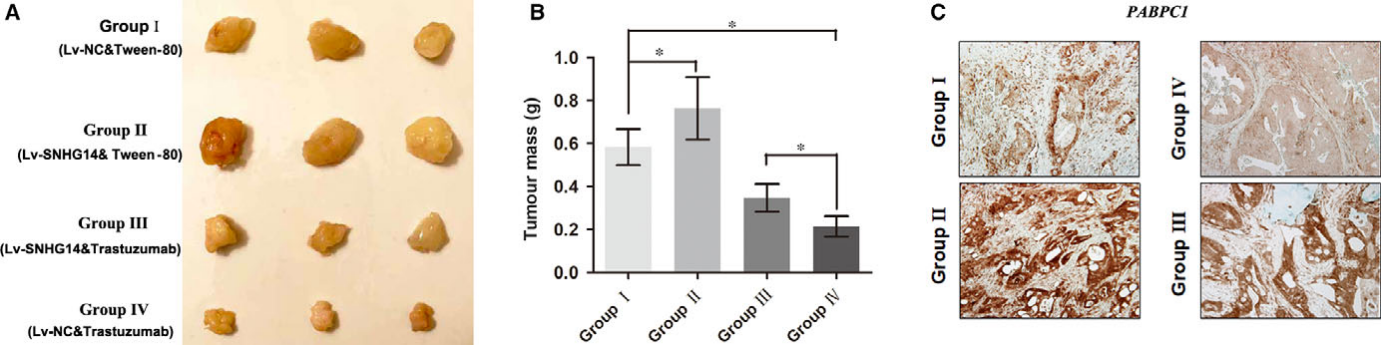
lncRNA SNHG14 promotes trastuzumab resistance in vivo. A, Photographs of tumours that developed in xenograft transplanted nude mouse tumour models treated orally with once daily 50 mg/kg trastuzumab or 1% Tween‐80 as control for 4 wk in different groups. B, Weights of tumours that developed in xenografts from different groups are shown. C, IHC analysis of expression levels of *PABPC1* in respective groups. *P <* *.05

To validate the in vitro data of lncRNA SNHG14, we established a model of nude mice bearing BT474 xenograft. BT474 parental cells that stably transfected with Lv‐SNHG14 or negative control Lv‐NC were injected into the flanks of nude mice. After the tumours were established, mice injected with both kinds of BT474 cells were treated orally with once daily 50 mg/kg trastuzumab or 1% Tween‐80 as control for 4 weeks. Herein, four mice xenograft treatment groups were established: Group I (Lv‐NC‐transfected cells + 1% Tween‐80), Group II (Lv‐SNHG14‐transfected cells + 1% Tween‐80), Group III (Lv‐SNHG14‐transfected cells + trastuzumab treatment) and Group IV (Lv‐NC‐transfected cells + trastuzumab treatment). More than three mice in each group were remained after excluding mice that were dead or with complications, such as skin necrosis due to infection. Tumours were stripped, and tumour mass was quantified (Figure 6A). The results showed SNHG14 promoted tumour growth in this xenograft model (Group II vs Group I), and trastuzumab treatment significantly suppressed the growth of tumour cells when compared with control groups (Group IV vs Group I). More importantly, with treatment of trastuzumab, tumour cells that infected with Lv‐SNHG14 grew faster than controls (Group III vs Group IV), suggesting that SNHG14 repressed the cell cytotoxicity induced by treatment with trastuzumab in vivo (Figure 6B). In addition, immunohistochemistry (IHC) analysis was conducted to determine whether SNHG14 affects the expression of *PABPC1* in xenograft tumour tissues. As shown in Figure 6C, overexpression of SNHG14 promoted the level of *PABPC1* in either 1% Tween‐80 or trastuzumab treatment group (Group II vs Group I or Group III vs Group IV, respectively), indicating that SNHG14 regulates breast cancer carcinogenesis and trastuzumab resistance via targeting *PABPC1*.

### 3.7 Nrf2 pathway is promoted by the up‐regulation of *PABPC1* induced by lncRNA SNHG14

**Figure 7:**
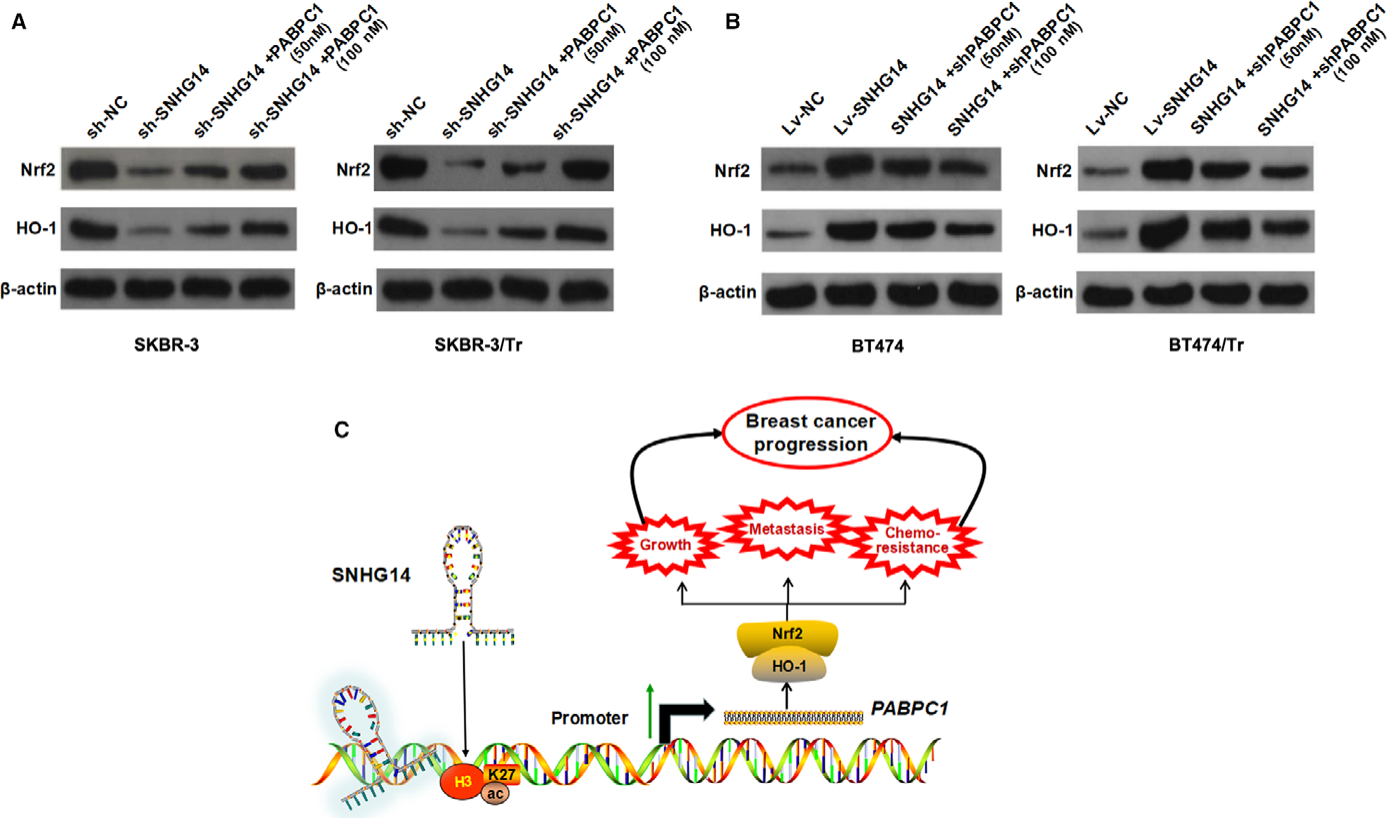
lncRNA SNHG14 activates Nfr2 signalling pathway depending on *PABPC1*. A, Western blot was performed to detect the protein level of Nrf2 and HO‐1 in SNHG14‐silenced SKBR‐3 and SKBR‐3/Tr cells transfected with Lv‐PABPC1. B, Western blot was performed to detect the protein level of Nrf2 and HO‐1 in SNHG14‐overexpressed BT474 and BT474/Tr cells transfected with PABPC1 shRNA. C, A schematic diagram of the role of lncRNA SNHG14 in breast cancer tumorigenesis and chemo‐resistance

To determine the signalling pathway that directly involved in breast cancer progression and chemo‐resistance, we used Signal Reporter Array to simultaneously investigate the activity changes of canonical signalling pathways in BT474 cells upon overexpression of SNHG14. The five most activated and five most silenced downstream pathways are shown in Table S2, among which we identified the Nrf2 pathway as the mostly activated one. Previous literatures suggested that Nrf2 pathway is one of the major signalling cascades involved in cell defense and survival against endogenous and exogenous stress, such as chemotherapy drugs.^20^ Therefore, we has been suggested that SNHG14 may participate in breast cancer tumorigenesis via regulating Nrf2 pathway depending on *PABPC1*. Western blot assay was performed to test this hypothesis, and we found that Nrf2 and HO‐1 were decreased in SKBR‐3 and SKBR‐3/Tr cells transfected with sh‐SNHG14 plasmids compared to negative control cells, while ectopic expression of *PABPC1* partially rescued this effect (Figure 7A). Conversely, Nrf2 and HO‐1 were increased in BT474 and BT474/Tr cells transfected with Lv‐SNHG14 compared to negative control cells, while silence of *PABPC1* partially diminished this promotion induced by SNHG14 overexpression (Figure 7B). To this end, we validated that the Nrf2 pathway is responsible for the SNHG14/PABPC1‐induced breast cancer progression.

## 4 DISCUSSION

Extensive efforts in the past have contributed to the understanding of both molecular and cellular mechanisms of action of chemo‐resistance, one of the major causes for the failure of treatment with advanced cancer types. However, little progress has been made ever since.^21^ Thus, novel molecular signatures seem to hold great promise in tumour characterization and could be used as potential prognostic markers and treatment target. To identify potential molecular therapeutic markers for trastuzumab treatment, the functional relevance between lncRNA SNHG14 and breast cancer progression and trastuzumab resistance were investigated. Our own date showed that SNHG14 promoted breast cancer tumorigenesis and chemo‐resistance via activating *PABPC1* through H3K27 acetylation (Figure 7C).

The roles of lncRNAs in breast cancer progression have long been researched and SNHG14 was identified as an oncogene. In this study, we identified the expression level and functional role of SNHG14 in breast cancer progression and trastuzumab resistance. Breast cancer patients overexpressed with HER2 are associated with poor prognosis.^22^ HER2‐targeted therapy for HER2‐positive breast cancer patients has dramatically improved the survival rate and reduced mortality in recent history.^23^ HER2 gene amplification was first associated with worse clinical outcomes in the late 1980s by Slamon et al, and the following serious of studies revealed that residents at Asian‐Pacific areas were with high occurrence rate to be diagnosed with HER2‐positive status with a poorer diagnosis in comparison with other regions.^24^, ^25^, ^26^ Trastuzumab has proven efficacy as first‐line treatment of advanced HER2^+^ breast cancer patients. However, initial benefit lasts not long before the occurrence of conversion, resulting in an acquired resistance.^27^ Therefore, breakthroughs were needed in the finding of effective therapeutic targets and overcome of acquired trastuzumab resistance, especially for patients with HER2^+^ sites.

To further investigate how SNHG14 functions in breast cancer cells, we determined the downstream targeted genes by conducting RNA‐pull down experiments followed by mass spectrometry. On this basis, we identified *PABPC1* as one putative target. Polyadenylate‐binding proteins (PABPs) are a special type of proteins that interact in a sequence‐specific fashion with single‐stranded poly (A) by RNA recognition motif (RRM). PABPs are classified into PABPC in the cytoplasm and PABPN1 in the nucleus.^28^ PABPC coating of the poly(A) tail is intuitively considered as protecting mRNA against degradation by exonucleases. It binds specifically to the poly(A) tail of mRNA in cytoplasmic, is required for poly(A) shortening, ribosome recruitment and translation initiation.^29^ Yet, the relation of PABPC with deadenylation is complex. This conserved gene family includes at least 3 functional proteins: *PABPC1*,* PABPC3* and *PABPC4*. *PABPC1* is known to be involved in mRNA translation and degradation,^30^, ^31^ and stabilizes the 5′ cap of mRNA. In addition, *PABPC1* plays the most important role in deadenylation and protects poly (A) tail by covering the poly (A) tail. Previous studies show that *PABPC1* interacts with *AGO2* and is responsible for the microRNA‐mediated gene silencing.^32^ In addition, *PABPC1* also directly participate in gastric cancer tumorigenesis by influencing cell proliferation.^33^ These studies strongly suggest that *PABPC1* may play an oncogenic role in cancer progression under the regulation of SNHG14. As expected, we confirmed that *PABPC1* was essential for SNHG14‐induced breast cancer tumorigenesis and trastuzumab resistance.

We further explored the underlying regulation model of increased *PABPC1* by SNHG14. Mechanisms that generate transcript diversity are of fundamental importance in cancers. In recent years, regulatory factors such as histone modifications, suggesting that epigenetic features may have the ability not only to determine when and in which tissues certain genes are expressed, but also to influence how these transcripts are processed.^34^ Histone acetylation is a major histone modification involved in the regulation of chromatin structure and transcription. It neutralizes the positive charge on the lysine side chain, relaxing the chromatin structure and enhancing transcriptional activity.^35^ Histone acetylation named H3K27Ac was first discovered in yeast^36^ and recent advancements in DNA sequencing technology have enabled the analysis of histone acetylation distribution patterns through whole genome.^37^ In this study, we investigated the H3K27ac concentration at the promoter of *PABPC1* by analysis of ENCODE database, followed by a serious of experimental verifications including RT‐qPCR, ChIP and Western blots using breast cancer tissues and cells. We identified that *PABPC1* was highly enriched with H3K27ac at the promoter of PABPC1 in breast cancer tissues. Moreover, this histone acetylation was mediated by SNHG14 as evidenced by the changed enrichment induced by overexpression or knockdown of SNHG14 in breast cancer cells.

We should address some points which need a more comprehensive investigation in future studies. First, it is still unclear whether SNHG14 directly mediates the histone acetylation or by binding with the RNA‐binding proteins (RBPs), such as CREB‐binding protein (CBP), which is a transcriptional co‐activator with histone acetyltransferase (HAT) activity. Second, this study initially identified PABPC1 as a target protein through screening for SNHG14‐interacting proteins using RNA‐pulldown assay. However, we eventually verified that SNHG14 regulated the expression of PABPC1 at transcript level through mediating H3K27 acetylation. In the future, we need to reveal the underlying regulatory mechanism by which PABPC1 regulates SNHG14 expression at both transcript and protein levels.

Finally, we performed in vivo experiments to verify the in vitro results. As expected, SNHG14 abrogated the trastuzumab‐induced growth suppression of xenograft tumour, indicating that SNHG14 promotes trastuzumab resistance in our mice models. Moreover, SNHG14 promotes breast cancer tumorigenesis via regulating Nfr2 pathway depending on *PABPC1*. In summary, our study revealed that lncRNA SNHG14 promotes breast cancer progression and chemo‐resistance to trastuzumab treatment both in vitro and in vivo. Therefore, SNHG14 could be considered as a promising diagnostic biomarker and therapeutic target for breast cancer patients, enhancing the clinical benefits of trastuzumab therapy.

## CONFLICT OF INTEREST

The authors declare that they have no competing interests.
