# Supplementary material for: Long non‐coding RNA SNHG14 induces trastuzumab resistance of breast cancer via regulating PABPC1 expression through H3K27 acetylation
Source: J Cell Mol Med. 2018 Jul 31;22(10):4935–47. doi: 10.1111/jcmm.13758 (PMC6156344; doi:10.1111/jcmm.13758)
Supplement: Supplementary file 1 [file JCMM-22-4935-s001.doc]

Supplementary Table 1: Identification of SNHG14 binding proteins by MS.

| **Protein** | **Beads** | **SNHG14** | **Ratio (SNHG14/Beads)** |
| --- | --- | --- | --- |
| **PABPC1** | **0** | **3** | **NA** |
| U2AF1 | 1 | 3 | 3 |
| NKRF | 0 | 3 | NA |
| EF1D | 0 | 3 | NA |
| AIMP2 | 0 | 3 | NA |
| ROA0 | 0 | 3 | NA |
| RO60 | 0 | 3 | NA |
| ARP2 | 0 | 3 | NA |
| STT3B | 0 | 3 | NA |
| PCH2 | 1 | 3 | 3 |
| MRP1 | 0 | 3 | NA |
| LAS1L | 0 | 3 | NA |
| ARF6 | 1 | 3 | 3 |
| PLST | 0 | 3 | NA |
| PSAL | 0 | 3 | NA |
| TTL12 | 0 | 3 | NA |
| ERLN1 | 0 | 3 | NA |
| NSF | 0 | 3 | NA |
| AKAP8 | 0 | 3 | NA |
| GSTO1 | 0 | 3 | NA |
| AP1B1 | 0 | 3 | NA |
| DPM1 | 0 | 3 | NA |
| PSDE | 1 | 3 | 3 |
| KTN1 | 1 | 3 | 3 |

Beads: spectral counts of proteins in beads only group;

SNHG14: spectral counts of proteins in SNHG14 group;

Ratio (SNHG14/Beads): spectral count ratio of proteins comparing SNHG14 group to beads only group;

NA: not available.
