# Supplementary material for: Long non‐coding RNA SNHG14 induces trastuzumab resistance of breast cancer via regulating PABPC1 expression through H3K27 acetylation
Source: J Cell Mol Med. 2018 Jul 31;22(10):4935–47. doi: 10.1111/jcmm.13758 (PMC6156344; doi:10.1111/jcmm.13758)
Supplement: Supplementary file 2 [file JCMM-22-4935-s002.doc]

**Supplementary Table 2: Fold changes for the activities of different signaling reporters upon overexpression of SNHG14**

| **Reporters** | **Regulation** | **Fold change** | |
| --- | --- | --- | --- |
| Nrf2  mTOR  Bcl-2/Bax  MAPK/JNK  p53 | Up  Up  Up  Up  Up | 8.3  5.6  4.9  4.1  3.6 |  |
| NF-κB  Sp1  TCF/LEF  SMAD  FOXO | Down  Down  Down  Down  Down | 5.9  4.0  3.1  2.8  2.5 |  |
